# Radiomics and blood biomarkers for predicting efficacy of sintilimab plus lenvatinib in advanced hepatocellular carcinoma

**DOI:** 10.3389/fimmu.2026.1782008

**Published:** 2026-03-27

**Authors:** Yong Cui, Lijun Wang, Xiaoting Li, Kun Wang, Hongwei Wang, Quan Bao, Haitao Zhu, Xiaolei Gu, Qian Xing, Kemin Jin, Yingshi Sun, Baocai Xing

**Affiliations:** 1 Key Laboratory of Carcinogenesis and Translational Research (Ministry of Education), Department of Radiology, Peking University Cancer Hospital & Institute, Beijing, China; 2 Peking University Cancer Hospital (Inner Mongolia Campus)/Affiliated Cancer Hospital of Inner Mongolia Medical University, Department of Radiology, Inner Mongolia Cancer Center, Hohhot, China; 3 Key Laboratory of Carcinogenesis and Translational Research (Ministry of Education), Department of Hepatopancreatobiliary Surgery Unit I, Peking University Cancer Hospital & Institute, Beijing, China

**Keywords:** CT imaging, hepatocellular carcinoma, lenvatinib, predictive model, radiomics, sintilimab, systemic immune-inflammation index

## Abstract

**Background:**

Immune checkpoint inhibitor–based combination therapy has emerged as an important treatment option for advanced hepatocellular carcinoma (HCC), yet therapeutic response remains highly heterogeneous. Biomarkers that jointly reflect tumor-intrinsic heterogeneity and host immune status are needed to improve response stratification and better understand variability in immunotherapy outcomes.

**Methods:**

We evaluated the predictive value of Computed Tomography (CT) -derived radiomic heterogeneity and systemic inflammatory biomarkers in patients with advanced HCC treated with sintilimab plus lenvatinib. A total of 62 patients were included and divided into a training cohort (n = 36) and an independent real-world validation cohort (n = 26). Radiomic features were extracted from multiphase contrast-enhanced CT and summarized as a radiomics score. Hematological indices reflecting systemic immune-inflammatory status were assessed in parallel. An integrated model combining imaging-derived heterogeneity and immune-inflammatory markers was constructed for response stratification.

**Results:**

The radiomics score discriminated responders from non-responders with area under the curve (AUC) values of 0.840 in the training cohort and 0.759 in the validation cohort. Among systemic biomarkers, the systemic immune-inflammation index (SII) was independently associated with treatment response. The integrated model combining radiomic heterogeneity and SII demonstrated improved discriminatory performance (AUC 0.938 and 0.819 in training and validation cohorts, respectively). Stratification based on the combined model was associated with differences in event-free survival, supporting the biological relevance of multimodal immune–tumor characterization.

**Conclusions:**

Integrating imaging-derived tumor heterogeneity with systemic immune-inflammatory status may provide a biologically informed and non-invasive strategy for exploratory response stratification in advanced HCC undergoing immune checkpoint inhibitor–based therapy. Larger multicenter studies incorporating prospective validation and immune profiling are warranted to confirm clinical applicability.

## Introduction

Hepatocellular carcinoma remains one of the leading causes of cancer-related mortality worldwide and is frequently diagnosed at an advanced stage, precluding curative interventions (1–3). In recent years, immune checkpoint inhibitors targeting programmed cell death protein 1 (PD-1), particularly in combination with anti-angiogenic agents such as lenvatinib, have expanded therapeutic options for advanced HCC (4). Clinical trials and real-world studies have reported objective response rates of approximately 30–40%; however, durable benefit is observed only in a subset of patients, underscoring marked interindividual variability in immunotherapy response (5, 6).

From an immunological standpoint, response to immune checkpoint inhibitor–based therapy is shaped by the dynamic interplay between tumor-intrinsic characteristics and host immune context. Intratumoral heterogeneity, vascular architecture, and stromal composition influence immune cell infiltration and the establishment of an immunologically permissive or suppressive microenvironment. Concurrently, systemic immune and inflammatory status modulates antitumor immunity and may determine the capacity of immune checkpoint blockade to restore effective cytotoxic T-cell function (7, 8). Despite increasing recognition of these dual determinants, clinically accessible biomarkers capable of capturing both tumor-local and host-systemic immune factors remain limited.

Radiomics (9) has emerged as a non-invasive approach to quantify tumor phenotypic heterogeneity through high-dimensional feature extraction from medical imaging. In HCC, CT-derived radiomic features have been associated with tumor vascular patterns, hypoxia-related characteristics, and microenvironmental complexity—biological processes closely linked to immune responsiveness (10–13). Nevertheless, most radiomics-based predictive models focus primarily on tumor-intrinsic imaging signatures without incorporating host immune-related information, potentially limiting their biological interpretability in the context of immunotherapy.

In parallel, hematological biomarkers reflecting systemic immune-inflammatory status have gained attention as accessible predictors of immunotherapy outcomes (14–16). Indices such as the SII (17), which integrates neutrophil, platelet, and lymphocyte counts, are considered surrogate markers of the balance between pro-tumor inflammation and antitumor immune surveillance. Elevated neutrophil and platelet levels may contribute to immunosuppressive signaling and tumor progression, whereas reduced lymphocyte counts may reflect impaired adaptive immune responses. However, when used alone, these systemic markers lack tumor-specific spatial context and may insufficiently characterize intratumoral immune heterogeneity.

Given these complementary strengths and limitations, integrating imaging-derived tumor heterogeneity with systemic immune-inflammatory indicators may provide a more comprehensive framework for understanding variability in immunotherapy response. Conceptually, such an approach aims to approximate tumor–host immune interactions by combining spatially resolved tumor phenotypes with systemic immune context.

Accordingly, this study sought to evaluate the predictive value of CT-based radiomics features and pre-treatment hematological biomarkers in patients with advanced HCC treated with sintilimab plus lenvatinib. By developing and validating an integrated multimodal model, we aimed to explore whether combining tumor-intrinsic imaging heterogeneity with systemic immune status could enhance exploratory response stratification in the immunotherapy setting.

## Materials and methods

### Study design and patient population

This study employed a retrospective–prospective design to evaluate imaging-derived tumor heterogeneity and systemic immune-inflammatory biomarkers associated with treatment response in patients with advanced HCC receiving immune checkpoint inhibitor–based combination therapy.

The training cohort consisted of patients enrolled in a prospective clinical trial conducted at Peking University Cancer Hospital (ClinicalTrials.gov identifier: NCT04042805) evaluating sintilimab combined with lenvatinib in locally advanced HCC. The independent validation cohort included consecutive patients treated at the same institution under routine clinical practice during or after the trial period who received the same therapeutic regimen. The sample size reflects the number of eligible patients treated during the study period, and all analyses were conducted as exploratory investigations.

Inclusion criteria were: (1) Histologically or radiologically confirmed HCC; (2) Availability of baseline multiphase contrast-enhanced CT imaging; (3) Availability of pre-treatment hematological laboratory data;(4) Receipt of at least three cycles of sintilimab plus lenvatinib with evaluable response assessment. Exclusion criteria included:(1) Prior systemic therapy or locoregional treatment;(2) Non-standardized CT protocols unsuitable for radiomics analysis;(3) Missing imaging or laboratory data;(4) Loss to follow-up.

All patients received sintilimab (200 mg every 3 weeks) combined with lenvatinib (12 mg daily for body weight ≥60 kg or 8 mg daily for <60 kg). The study was approved by the institutional ethics committee and conducted in accordance with the Declaration of Helsinki. Written informed consent was obtained from all participants.

### CT acquisition and response assessment

Baseline multiphase contrast-enhanced CT scans were performed prior to treatment initiation using multi-detector CT scanners (Philips and GE Healthcare platforms). Imaging protocols included non-contrast, arterial phase (20–30 seconds), portal venous phase (70–80 seconds), and delayed phase acquisitions.

Treatment response was evaluated every 9 weeks during the first year and every 12 weeks thereafter, according to the modified Response Evaluation Criteria in Solid Tumors (mRECIST). Patients achieving complete response or partial response were classified as responders, while those with stable or progressive disease were classified as non-responders.

### Imaging indicator analysis

Prior to analysis, all images were reviewed by two independent radiologists (XL Gu and Y Cui) to ensure technical adequacy. Imaging features were categorized into two groups: subjective and objective.

Subjective imaging features included qualitative tumor characteristics assessed by the radiologists, such as the proximity of the tumor to the liver capsule, the presence of large tumor vessels within the lesion, arterial phase enhancement, visibility of the peripheral tumor outline, capsule integrity, regularity of tumor margins during the portal venous phase, homogeneity of enhancement in the portal venous phase, surrounding enhancement during the arterial phase, and evidence of necrosis, cystic degeneration, or intratumoral hemorrhage.

Objective imaging features included radiomics features, which were quantitatively extracted using specialized software. Tumor regions of interest were manually delineated on baseline CT images using 3D Slicer software (version 4.9.0, https://www.slicer.org). Segmentation was performed on non-contrast, arterial, and portal venous phase images by an experienced radiologist blinded to clinical outcomes. To assess intra-observer reproducibility, repeated segmentation was performed in a subset of patients after a one-month interval. Intraclass correlation coefficients (ICC) were calculated, and features with ICC < 0.75 were excluded from further analysis to ensure robustness.

Radiomics features were extracted using the PyRadiomics package (version 3.1.0) implemented in Python. A total of 107 features were extracted for each CT phase, including 18 first-order features, 24 gray-level co-occurrence matrix (GLCM) features, 14 gray-level dependence matrix (GLDM) features, 16 gray-level run length matrix (GLRLM) features, 16 gray-level size zone matrix (GLSZM) features, 5 neighboring gray-tone difference matrix (NGTDM) features, and 14 shape features. In total, 321 features were extracted across the three CT phases. No ComBat harmonization was applied, which is acknowledged as a methodological limitation.

Feature selection was conducted exclusively within the training cohort to avoid data leakage. Initially, features significantly associated with treatment response were identified using univariable analysis. Redundant features were subsequently removed based on correlation analysis to reduce multicollinearity. Following feature selection, three machine learning classifiers—support vector machine (SVM), extreme gradient boosting (XGBoost) and least absolute shrinkage and selection operator (LASSO) logistic regression—were used to construct predictive models. Each classifier generated a Radiomics Score, which estimated the probability of treatment response for individual patients. To mitigate class imbalance within the training set, the Synthetic Minority Over-sampling Technique (SMOTE) was applied during model training only. The independent validation cohort was evaluated without oversampling.

### Hematological indicator analysis

Peripheral blood samples were collected within one week prior to treatment initiation. Hematological indices reflecting systemic immune and inflammatory status were calculated, including neutrophil-to-lymphocyte ratio (NLR), platelet-to-lymphocyte ratio (PLR), lymphocyte-to-monocyte ratio (LMR), SII and systemic inflammation response index (SIRI). The NLR was calculated by dividing the neutrophil count by the lymphocyte count, while the PLR was calculated by dividing the platelet count by the lymphocyte count. The LMR was calculated by dividing the lymphocyte count by the monocyte count. The SII was computed by multiplying the platelet count and neutrophil count, then dividing by the lymphocyte count. Similarly, the SIRI was calculated by multiplying the neutrophil count and monocyte count, then dividing by the lymphocyte count. These markers were evaluated as potential indicators of host immune-inflammatory status relevant to immunotherapy response.

### The combination model construction, evaluation and validation

Subjective imaging features, hematological indicators, and other potential clinical factors were assessed using the Chi-square test or Fisher’s exact test. Features with a significance level of less than 0.05, along with the radiomics score, were incorporated into a logistic regression model to construct the combination model using a machine learning approach. The diagnostic performance of the model was assessed using the receiver operating characteristic (ROC) curve. To evaluate the clinical relevance of the model, survival analysis was conducted on the model-predicted response groups using Kaplan–Meier curves and log-rank testing.

### Statistical analysis

All analyses were exploratory, and no adjustment for multiple testing was performed given the hypothesis-generating nature of the study. A sample of 13 from the responder group and 13 from the non-responder group achieves 80% power to detect a difference of 0.25 between the area under the ROC curve under the null hypothesis of 0.7 and an AUC under the alternative hypothesis of 0.95 using a two-sided z-test at a significance level of 0.05. Thus at least 26 patients were needed for the validation group. Continuous variables with a normal distribution were presented as means with standard deviations, while categorical variables were represented as counts. Comparisons of continuous variables between response groups were made using either Student’s t-test or the Mann–Whitney U test as appropriate. Categorical variables were compared using the chi-square test or Fisher’s exact test. Kaplan–Meier analysis and log-rank testing were performed to explore event-free survival (EFS) differences between model-stratified groups. A two-sided P value < 0.05 was considered statistically significant. Statistical analyses were conducted using R4.4.2 (R Core Team, 2024) and SPSS 25.0 (SPSS, Chicago, IL) software.

## Results

### Patient characteristics

Between August 1, 2019, and December 10, 2021, a total of 62 HCC patients were included, with 36 assigned to the training cohort and 26 to the independent validation cohort. As of the data cutoff date (September 30, 2024), 12 patients in the training set had undergone surgery with curative intent, and 1 patient had received a combination of radiofrequency ablation and stereotactic radiotherapy. In the validation set, 6 patients had undergone surgery. The remaining patients in both cohorts continued to receive the combination therapy until tumor progression, adverse reactions became intolerable, or they withdrew informed consent.

Based on the mRECIST, 24 patients (66.7%) in the training set and 16 patients (61.5%) in the validation set were classified as responders. **Table 1** presents the baseline characteristics of the patients. All variables were comparable between the training and validation cohorts, with the exception of three subjective radiomics features: surrounding enhancement in the arterial phase, necrosis, and cystic changes. Moderate to excellent ICC (0.481 to 1.000) was achieved for the CT variables, as detailed in **Supplementary Table 1**.

**Table 1:** Comparison of Baseline Characteristics Between the Training and Validation Cohorts.

Variables	Category	Training cohort			Validation cohort			P’
		Responder (n=24)	Non-responder (n=12)	P	Responder (n=16)	Non-responder (n=10)	P	
General clinical data:								
Gender	Female	1	4	0.034	2	2	0.625	>0.999
	Male	23	8		14	8		
Age (years)		58 ± 12	60 ± 10	0.685	61 ± 11	63 ± 11	0.642	0.325
ECOG, PS	0	17	7	0.479	11	9	0.352	0.821
	1	7	5		5	1		
BCLC Stage	B	11	6	0.813	3	5	0.189	0.193
	C	13	6		13	5		
Etiology of HCC	HBV	22	11	1.000	15	10	1.000	1.000
	HCV/Other	2	1		1	0		
Largest tumor diameter (mm)		87 ± 36	101 ± 49	0.338	78 ± 48	64 ± 33	0.42	0.088
Number of tumors	Single	16	7	0.72	6	4	>0.999	0.048
	Multiple	8	5		10	6		
AFP (ng/ml)	<400	15	6	0.473	7	7	0.248	0.725
	≥400	9	9		9	3		
Subjective radiomics features of tumors:								
Near the liver capsule	No	3	0	>0.999	3	1	>0.999	0.439
	Yes	21	12		13	9		
Large tumor vessels inside the tumor	No	11	5	0.813	8	4	0.701	0.894
	yes	13	7		8	6		
Arterial phase enhancement	No	1	0	>0.999	2	1	>0.999	0.296
	Yes	23	12		14	9		
Peripheral outline	Unclear	3	0	0.536	2	1	>0.999	0.689
	Clear	21	12		14	9		
Capsule integrity	Complete	22	11	>0.999	16	10	NA	0.258
	Incomplete	2	1		0	0		
Irregular protrusions at the margin in the portal venous phase	No	16	2	0.005	9	3	0.191	0.829
	Yes	8	10		7	7		
Uneven enhancement in the portal venous phase	No	3	1	>0.999	3	2	>0.999	0.473
	Yes	21	11		13	8		
Surrounding enhancement in the arterial phase	No	23	11	>0.999	12	7	>0.999	0.028
	Yes	1	1		4	3		
Necrosis and cystic changes	No	9	6	0.473	11	8	0.668	0.014
	Yes	15	6		5	2		
Intratumoral hemorrhage	No	24	10	0.105	16	9	0.385	>0.999
	Yes	0	2		0	1		
Hematological indicators:								
NLR (Neutrophil-to-lymphocyte ratio)	<5	24	11	0.333	13	6	0.369	0.007
	≥5	0	1		3	4		
PLR (Platelet-to-lymphocyte ratio)	<200	22	9	0.307	12	8	>0.999	0.502
	≥200	2	3		4	2		
LMR (Lymphocyte-to-monocyte ratio)	<340	8	3	0.715	7	6	0.42	0.121
	≥340	16	9		9	4		
SII (Systemic Immune-Inflammation Index)	<340	16	3	0.018	8	2	0.126	0.549
	≥340	8	9		8	8		
SIRI (Systemic Inflammation Response Index)	<1	16	6	0.471	10	3	0.107	0.384
	≥1	8	6		6	7		

P, comparison between responder and non-responder groups; P’, comparison between training and validation groups; BCLC, Barcelona Clinic Liver Cancer; ECOG PS, Eastern Cooperative Oncology Group Performance Status; HBV, hepatitis B virus; HCC, hepatocellular carcinoma; HCV, hepatitis C virus.

### Radiomics model performance

**Figure 1:**
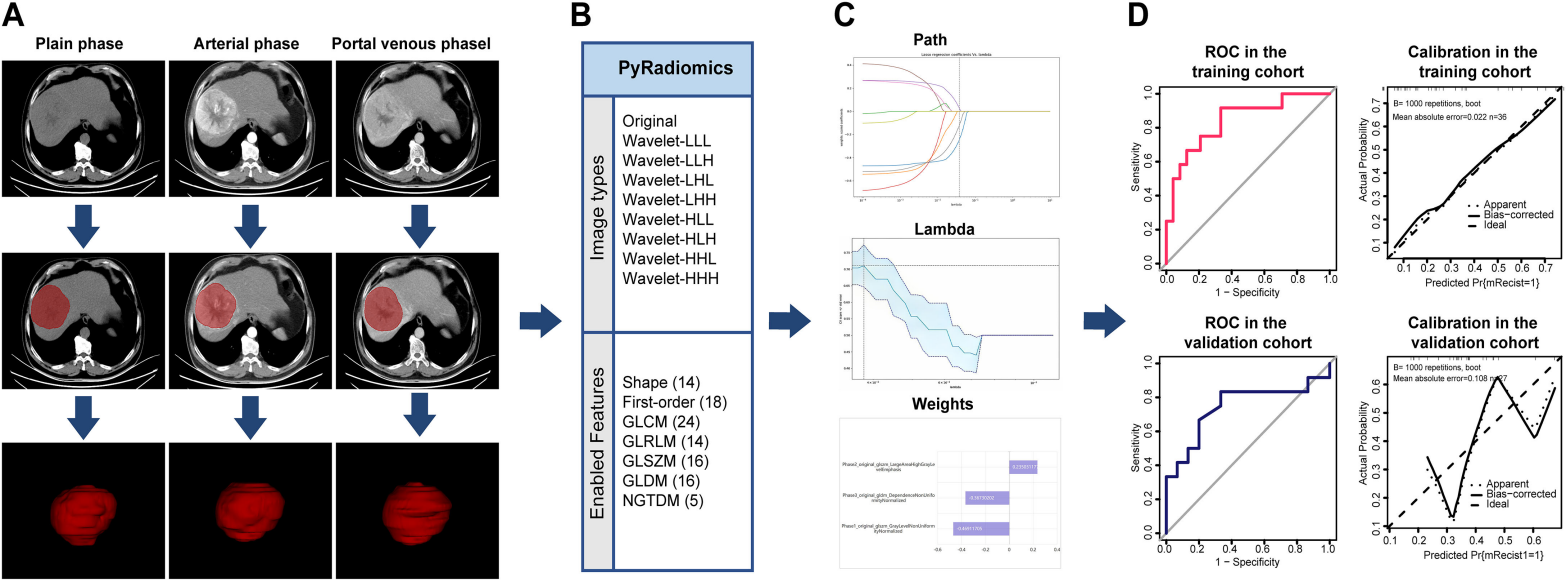
Radiomics workflow for model development and validation **(A)** Imaging Segmentation: Multiphase contrast-enhanced CT images (plain, arterial, and portal venous phases) were acquired, and regions of interest (ROIs) were manually delineated. **(B)** Feature Extraction: A total of 107 radiomic features, including shape, first-order, and texture features (GLCM, GLRLM, GLSZM, GLDM, NGTDM), were extracted from both original and wavelet-transformed images using PyRadiomics. **(C)** Feature Selection: The LASSO regression with cross-validation was employed to select the most predictive features. **(D)** Model Evaluation: ROC curves assessed discrimination performance, while calibration curves evaluated model accuracy in both training and validation cohorts.

Initially, a radiomics model based solely on objective imaging features was developed to predict treatment efficacy. Specifically, three CT-based radiomics models were constructed using the LASSO, SVM, and XGBoost algorithms. The diagnostic performance of these models in both the training and validation cohorts is presented in **Table 2**. Among the evaluated classification approaches, the LASSO-based radiomics model demonstrated stable performance and was selected for further analysis. Performance in the validation cohort was evaluated without oversampling. The radiomics workflow is illustrated in **Figure 1**.

**Table 2:** Diagnostic performance of three radiomics models in the training and validation cohorts.

Methods	Training	Validation
LASSO	0.840 (95%CI,0.701 to 0.980)	0.750 (95%CI,0.544 to 0.956)
VM	0.854 (95%CI,0.7109 to 0.989)	0.600 (95%CI,0.373 to 0.827)
XGBoost	0.898 (95%CI,0.797 to 0.998)	0.672 (95%CI,0.458 to 0.887)

### Associations of hematological biomarkers with treatment response

Among the evaluated hematological markers, the SII was significantly associated with treatment response (P = 0.018). Patients with elevated SII were more frequently classified as non-responders. Other inflammatory indices did not show statistically significant associations.

### The combination model construction, evaluation, and validation

**Figure 2:**
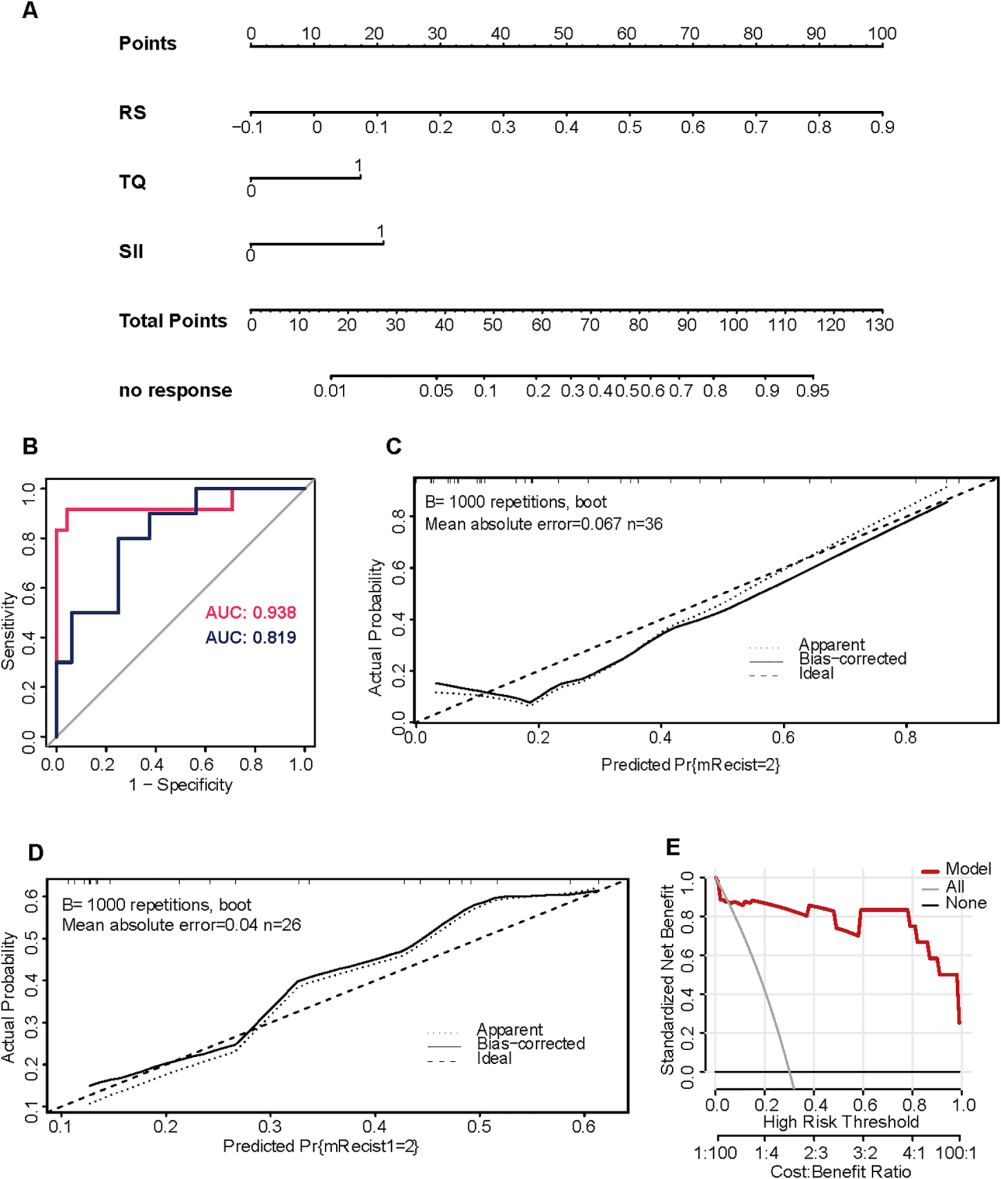
Combination model development and validation **(A)** Nomogram Construction: A model combining the radiomics score (RS) and SII was developed to predict treatment non-response. **(B)** ROC Analysis: The model achieved an AUC of 0.938 in the training cohort and maintained good performance in the validation cohort with an AUC of 0.819. **(C, D)** Calibration Curves: Calibration curves showed strong agreement between predicted and actual probabilities, with mean absolute errors of 0.04 and 0.067. **(E)** Decision Curve Analysis: The model demonstrated superior clinical net benefit across threshold probabilities.

To enhance predictive accuracy, we integrated the radiomics score with significant clinical and hematological variables into a combination model. A multivariable logistic regression analysis using a stepwise selection method identified the radiomics score (OR = 4408.23, P = 0.028) and the SII-based classification (OR = 10.30, P = 0.048) as independent predictors of treatment non-response. Additionally, the presence of tumor margin protrusions during the portal venous phase showed a trend toward statistical significance (OR = 5.94, P = 0.089). The combined model achieved AUCs of 0.938 in the training cohort and 0.819 in the validation cohort, demonstrating improved discrimination compared with single-modality models as detailed in **Table 3** and **Figure 2**. Calibration analysis indicated good agreement between predicted and observed probabilities. Decision curve analysis suggested potential clinical net benefit across a range of threshold probabilities.

**Table 3:** Diagnostic performance of the radiomics score, subjective CT imaging features, hematological biomarkers, and the combined model in the training and validation cohorts.

Model/Feature	Training					Validation			
Model/Feature	AUC	Cutoff	Sen	Spe	Acu	AUC	Sen	Spe	Acu
Radiomics score	0.840	>0.37	0.92	0.67	0.78	0.759	0.4	1	0.77
Irregular protrusions at the tumor edge	0.750	Yes	0.83	0.63	0.69	0.650	0.80	0.56	0.65
SII classification	0.708	>344	0.75	0.63	0.67	0.667	0.70	0.63	0.65
Combination model	0.938	>0.45	0.92	0.96	0.94	0.819	0.8	0.75	0.77

AUC, area under curve; Sen, sensitivity; Spe, specificity; Acu, accuracy; SII, Systemic immune-inflammation index.

### Exploratory survival analysis

The median EFS was 17 months (range: 2–61 months) in the training cohort, and 18 months (range: 2–51 months) in the validation cohort. Patients were categorized into model-predicted non-responder and responder groups based on the cutoff value determined by the combination model.

**Figure 3:**
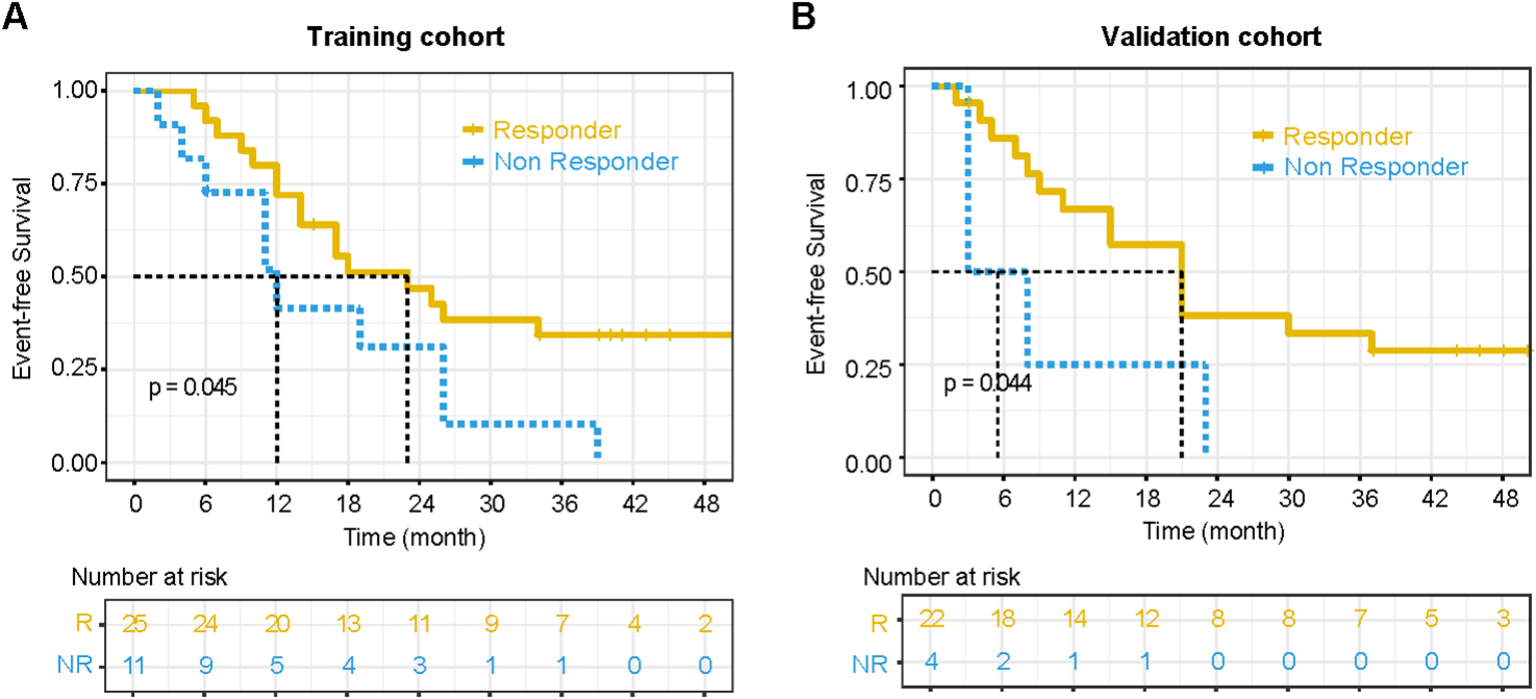
Kaplan–meier survival analysis based on the combination model patients were classified into responder and non-responder groups based on the model, confirming its prognostic value. **(A)** Training Cohort: Non-responders had significantly worse event-free survival (EFS) compared to responders (P = 0.045). **(B)** Validation Cohort: The non-responder group had inferior EFS relative to responders (P = 0.044).

In the training cohort, 24 patients were assigned to the model-predicted non-responder group, and 12 patients were assigned to the responder group. In the validation cohort, 14 patients were classified as non-responders and 12 patients as responders. The model-predicted non-responder group exhibited significantly inferior EFS compared to the responder group in both cohorts, with P-values of 0.045 in the training cohort and 0.044 in the validation cohort, as shown in **Figure 3**. These findings support the clinical relevance of multimodal response stratification, although the survival analysis was exploratory.

## Discussion

This study suggests that tumor-intrinsic imaging heterogeneity and systemic immune-inflammatory status jointly contribute to variability in response to immune checkpoint inhibitor–based therapy in advanced HCC. The principal finding is that tumor imaging heterogeneity and systemic immune-inflammatory status jointly contribute to treatment response stratification. Specifically, a CT-derived radiomics score and the SII independently correlated with therapeutic response, and their integration improved predictive performance compared with either modality alone.

Although radiomics is fundamentally an imaging-based technique, increasing evidence suggests that quantitative texture features may indirectly reflect tumor immune microenvironment characteristics. Prior studies (10–13) have linked radiomic heterogeneity patterns to immune cell infiltration, stromal composition, vascular remodeling, and hypoxic gradients—all of which influence responsiveness to immune checkpoint blockade. In this context, the radiomics score derived in our study may capture spatial variations in tumor architecture that parallel immune-related biological heterogeneity. Interestingly, irregular protrusions at the tumor margin in the portal venous phase did not reach statistical significance, although a trend toward association with poor response was observed. Such morphological irregularities have been previously associated with microvascular invasion (MVI) (18) and aggressive tumor behavior (19, 20). The lack of statistical significance in our cohort may be attributable to limited sample size or insufficient power. Nevertheless, this imaging phenotype warrants further evaluation in larger studies to clarify its potential predictive value.

Systemic immune-inflammatory status is increasingly recognized as a determinant of immunotherapy outcomes (21–23). The SII integrates neutrophil, platelet, and lymphocyte counts and reflects the dynamic balance between pro-tumor inflammatory signaling and anti-tumor adaptive immunity (17, 24). Elevated neutrophils and platelets may promote tumor progression through cytokine secretion, angiogenic factor release, and suppression of cytotoxic lymphocyte function, whereas lymphocyte depletion reflects impaired immune surveillance. Therefore, elevated SII may represent a systemic immune milieu unfavorable for effective immune checkpoint blockade, which is consistent with the poorer response observed in our cohort. Importantly, these hematological indices serve as indirect surrogates of immune dynamics rather than direct measurements of the tumor immune microenvironment.

Importantly, tumor-intrinsic heterogeneity and host systemic immune status represent two biologically interconnected dimensions of cancer progression. While radiomics-derived features may reflect localized immune–tumor interactions within the tumor microenvironment, hematological biomarkers such as SII capture systemic immune dynamics. The integration of these dimensions may provide a more comprehensive approximation of tumor–host immune interplay, which is particularly relevant in the context of immune checkpoint inhibitor–based therapies.

From a clinical standpoint, early identification of patients unlikely to respond to immune checkpoint inhibitor–based therapy remains a critical challenge. A non-invasive model integrating imaging-derived heterogeneity and systemic immune markers may support risk stratification and closer monitoring. However, the present findings remain exploratory and hypothesis-generating, and prospective validation is required before implementation in treatment decision-making.

Several limitations should be acknowledged. First, the sample size was modest, particularly for non-responder cases, which increases the risk of model instability and overfitting. Although an independent validation cohort was included, both cohorts originated from the same institution, and therefore the validation represents a real-world split-sample validation rather than true external validation. Second, the radiomics feature selection strategy relied on univariate filtering and correlation-based reduction, which may introduce selection bias in small datasets. The absence of nested cross-validation or feature stability assessment further limits robustness. Third, synthetic oversampling was applied to address class imbalance. While this approach may improve classifier discrimination, it may also alter feature distributions and affect generalizability. Finally, the survival analysis based on model-predicted response groups was exploratory, as the model was trained to predict radiologic response rather than survival endpoints. Taken together, our findings suggest a dual-layer immune–tumor interaction framework, in which intratumoral structural heterogeneity and systemic inflammatory balance jointly modulate sensitivity to immune checkpoint inhibition.

## Conclusion

In conclusion, this study suggests that integrating CT-derived radiomic heterogeneity with systemic immune-inflammatory status may provide a biologically informed, hypothesis-generating framework for predicting response to immune checkpoint inhibitor–based combination therapy in advanced HCC. These findings support the concept that both tumor-intrinsic architecture and host immune dynamics contribute to therapeutic sensitivity. Future multicenter and prospective studies incorporating standardized imaging and immunological profiling are warranted to validate and refine this multimodal predictive strategy.

## Data Availability

The original contributions presented in the study are included in the article/**Supplementary Material**, further inquiries can be directed to the corresponding author/s.
