# Supplementary material for: Radiomics and blood biomarkers for predicting efficacy of sintilimab plus lenvatinib in advanced hepatocellular carcinoma
Source: Front Immunol. 2026 Mar 27;17:1782008. doi: 10.3389/fimmu.2026.1782008 (PMC13066827; doi:10.3389/fimmu.2026.1782008)
Supplement: Supplementary file 1 [file Table1.docx]

**Supplementary table 1.** [Intraclass Correlation Coefficient](https://www.baidu.com/link?url=dgLrb1he-Ku3dYK13xhTzta1Ot4vVjVV7ixVCHEX3Q-M5GhdKUSKVKldYyjq9z-4bWnEcvSM2UlnLCSw6BSIeq&wd=&eqid=fed863cd000575a20000000367c05642) for subjective CT variables

| Subjective radiomics features of tumors | ICC | evaluation |
| --- | --- | --- |
| Near the liver capsule | 1.000 | excellent |
| Large tumor vessels inside the tumor | 0.481 | moderate |
| Arterial phase enhancement | 0.512 | moderate |
| Peripheral outline | 0.787 | substantial |
| Capsule integrity | 1.000 | excellent |
| Irregular protrusions at the margin in the portal venous phase | 0.746 | substantial |
| Uneven enhancement in the portal venous phase | 0.567 | moderate |
| Surrounding enhancement in the arterial phase | 0.626 | substantial |
| Necrosis and cystic changes | 0.461 | moderate |
| Intratumoral hemorrhage | 1.000 | excellent |

ICC: intraclass correlation coefficient.
